# Early Life Adversity and Polygenic Risk for High Fasting Insulin Are Associated With Childhood Impulsivity

**DOI:** 10.3389/fnins.2021.704785

**Published:** 2021-09-01

**Authors:** Aashita Batra, Lawrence M. Chen, Zihan Wang, Carine Parent, Irina Pokhvisneva, Sachin Patel, Robert D. Levitan, Michael J. Meaney, Patricia Pelufo Silveira

**Affiliations:** ^1^Integrated Program in Neuroscience, McGill University, Montreal, QC, Canada; ^2^Douglas Mental Health University Institute, McGill University, Montreal, QC, Canada; ^3^Mood and Anxiety Disorders Program, Centre for Addiction and Mental Health, Toronto, ON, Canada; ^4^Ludmer Centre for Neuroinformatics and Mental Health, Douglas Research Centre, McGill University, Montreal, QC, Canada; ^5^Translational Neuroscience Programme, Singapore Institute for Clinical Sciences, Agency for Science, Technology and Research (A^∗^STAR), Singapore, Singapore

**Keywords:** ALSPAC, MAVAN, fasting insulin, impulsivity, early life adversity

## Abstract

While the co-morbidity between metabolic and psychiatric behaviors is well-established, the mechanisms are poorly understood, and exposure to early life adversity (ELA) is a common developmental risk factor. ELA is associated with altered insulin sensitivity and poor behavioral inhibition throughout life, which seems to contribute to the development of metabolic and psychiatric disturbances in the long term. We hypothesize that a genetic background associated with higher fasting insulin interacts with ELA to influence the development of executive functions (e.g., impulsivity in young children). We calculated the polygenic risk scores (PRSs) from the genome-wide association study (GWAS) of fasting insulin at different thresholds and identified the subset of single nucleotide polymorphisms (SNPs) that best predicted peripheral insulin levels in children from the Avon Longitudinal Study of Parents and Children (ALSPAC) cohort [*N* = 467; *p*_*t*– initial_ = 0.24 (10,296 SNPs), *p*_*t*– refined_ = 0.05 (57 SNPs)]. We then calculated the refined PRS (rPRS) for fasting insulin at this specific threshold in the children from the Maternal Adversity, Vulnerability and Neurodevelopment (MAVAN) cohort and investigated its interaction effect with adversity on an impulsivity task applied at 36 months. We found a significant effect of interaction between fasting insulin rPRS and adversity exposure predicting impulsivity measured by the Snack Delay Task at 36 months [β = −0.329, *p* = 0.024], such that higher PRS [β = −0.551, *p* = 0.009] was linked to more impulsivity in individuals exposed to more adversity. Enrichment analysis (MetaCore^TM^) of the SNPs that compose the fasting insulin rPRS at this threshold was significant for certain nervous system development processes including dopamine D2 receptor signaling. Additional enrichment analysis (FUMA) of the genes mapped from the SNPs in the fasting insulin rPRS showed enrichment with the accelerated cognitive decline GWAS. Therefore, the genetic background associated with risk for adult higher fasting insulin moderates the impact of early adversity on childhood impulsivity.

## Introduction

Early life adversity (ELA) increases the risk for adult chronic disease, including psychopathology, metabolic, endocrine, and cardio-metabolic conditions (Malaspina et al., 2008; Rice et al., 2010; O’Donnell and Meaney, 2017; Van den Bergh et al., 2017; McQuaid et al., 2019; Monk et al., 2019). Neuroimaging studies have associated certain prenatal adversities with altered structural and functional trajectories in brain development (Bock et al., 2015; Egeland et al., 2015; Kim et al., 2015; O’Donnell and Meaney, 2017; Osborne et al., 2018; Monk et al., 2019). Since certain areas of the brain continue developing until late adolescence, the brain is also highly sensitive to postnatal adversity. Childhood adversity has been linked to long term behavioral outcomes and neurobiological consequences: emotional problems (Danese et al., 2020), aggressive behaviors (El-Khodary and Samara, 2020), changes in brain electrical activity (Marshall et al., 2004; Vanderwert et al., 2010), cognitive functions (Burneo-Garcés et al., 2019), and executive functions (Ursache et al., 2016). The mechanisms contributing to the development of these phenotypes involve gene by environment interactions resulting in behavioral differences (e.g., attention, impulsivity, and food preferences). However, not all individuals exposed to adversity develop these alterations. Responses to early adversity exposure have individual differences that are mostly driven by the genetic background.

At the neuroendocrine level, ELA is linked to alterations in responsivity to stress while also altering insulin sensitivity at different ages. Stressful conditions or adversities happening early in life, either pre- or postnatally, can affect glucose homeostasis and insulin function in the short and long terms. Some adversities are associated with a higher risk for insulin resistance and diabetes, such as: maternal/paternal history of diabetes (Beck-Nielsen and Groop, 1994; Groop et al., 1996), exposure to gestational diabetes (Krishnaveni et al., 2005), socioeconomic status (Everson et al., 2002), placental insufficiency (Camacho et al., 2017), cigarette smoking (Mouhamed et al., 2016), maternal malnutrition (Reusens et al., 2011), and chronic stress (Han et al., 2016; Yan et al., 2016). Such adverse events associate with both growth and metabolism (Wijlaars et al., 2011; Bhopal et al., 2019; Salmela et al., 2019). These events also alter responses to subsequent stressors (McGowan et al., 2009; Labonte et al., 2012; Peckins et al., 2020) and induce chronic inflammation (Flouri et al., 2020; Kokosi et al., 2020), both of which modify glucose homeostasis and insulin sensitivity (Facchi et al., 2020; Zannas et al., 2020). Beyond acute effects on brain development and child behavior (Lim et al., 2018; Chen et al., 2019; Lambert et al., 2019), long-term effects of adversity increase the risk for both metabolic diseases (Thomas et al., 2008; Fuller-Rowell et al., 2019) as well as psychopathologies later in life (Vaughn-Coaxum et al., 2019; Lund et al., 2020; Vogel et al., 2020).

Insulin is one of the primary hormonal regulators of metabolism in animals with several different functional roles (Brockman and Laarveld, 1986). Although most peripheral tissues depend on insulin signaling to acquire glucose, such is not the case with the brain as insulin is not needed for glucose transport into neurons (Kullmann et al., 2016). However, brain insulin does play a role as a neuroregulatory peptide (Woods et al., 1979; Bruning et al., 2000; Hallschmid et al., 2012) acting in different brain areas such as the ventral tegmental area, striatum, hypothalamus, hippocampus, olfactory bulb, and prefrontal cortex (Ghasemi et al., 2013). Insulin within these areas modulates the development and expression of different executive function behaviors (Kullmann et al., 2016), such as attention, inhibitory control, and working memory. Insulin has also been shown to reduce activity in the prefrontal areas that control behaviors such as inhibitory control of eating (Heni et al., 2015). Furthermore, abnormal insulin levels and function are seen in Alzheimer’s patients where insulin impairments have been linked to learning deficits and memory formation impairments (Zhao and Alkon, 2001).

Considering that the genetic background represents variations in biological function, our objective was to develop a model that predicts an executive function behavior, impulsivity, as a function of the interaction between biological markers of elevated fasting insulin levels and ELA in children. To do so, we first assessed the relationship between a polygenic risk score (PRS) derived from genome-wide associations with high fasting insulin (Scott et al., 2012) and the actual peripheral insulin levels measured in children from the Avon Longitudinal Study of Parents and Children (ALSPAC) cohort (Boyd et al., 2013; Fraser et al., 2013). The genome-wide association study (GWAS) of fasting insulin (fasting insulin GWAS) was performed in adults where the insulin measured was collected from individuals following a fasting period (Scott et al., 2012). Since our study inspects the role of insulin in children, we used the ALSPAC cohort’s data on peripheral insulin levels to identify the polygenic markers most highly associated with peripheral insulin levels in children. We further refined these markers to only include single nucleotide polymorphisms (SNPs) that significantly predicted peripheral insulin levels in ALSPAC. Because brain insulin levels are not readily measured or available, a genetic marker reflecting peripheral insulin levels in children was used to inspect insulin’s role in neurodevelopmental behaviors. Using the SNPs identified in the discovery cohort ALSPAC, we calculated a refined PRS (rPRS) in an independent cohort [Maternal Adversity, Vulnerability and Neurodevelopment (MAVAN)] to investigate the interaction between the genetic background associated with fasting insulin in children and ELA to predict childhood impulsivity. Our methodology allowed us to create a PRS that is highly associated with fasting insulin in children (ALSPAC) and then investigate behavior outcomes in an independent cohort of children (MAVAN), thus refining a GWAS obtained by an adult cohort (Scott et al., 2012).

## Materials and Methods

### Participants

We used data from two prospective birth cohorts, one based in England (ALSPAC) (Northstone et al., 2019) and the other in Canada (MAVAN) (O’Donnell et al., 2014) to analyze the gene by environment interaction effects on cognitive neurodevelopment outcomes.

#### Avon Longitudinal Study of Parents and Children

**FIGURE 1:**
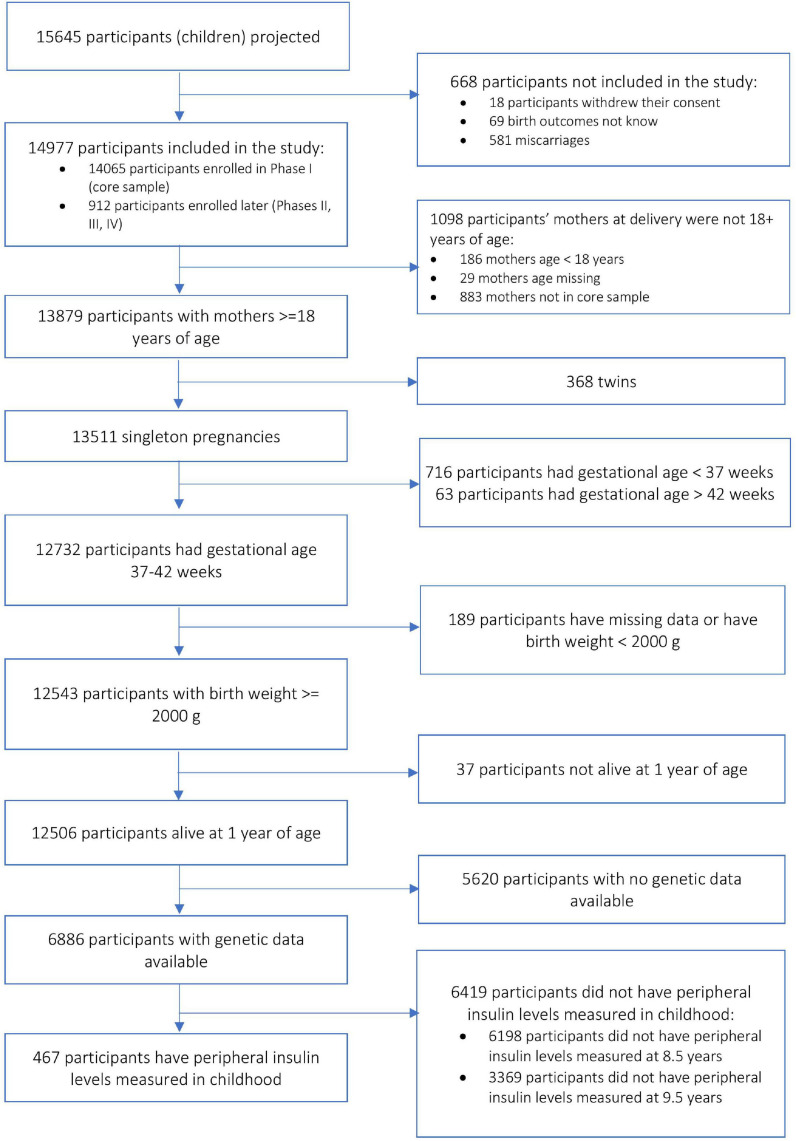
Block scheme describing sample selection in the ALSPAC cohort.

The ALSPAC cohort included pregnant women from the county of Avon, United Kingdom (Boyd et al., 2013; Fraser et al., 2013; Northstone et al., 2019) (*N* = 14,541) with expected delivery dates between April 1991 and December 1992. Additional recruitment (*N* = 913) was done during later phases, bringing the total sample size to 15,454. Participants provided informed written consent to participate in the study. Consent for biological samples had been collected in accordance with the Human Tissue Act (2004). Ethics approval for the study was obtained from the ALSPAC Ethics and Law Committee and the local research ethics committees (a full list of the ethics committees that approved different aspects of the ALSPAC studies is available at http://www.bristol.ac.uk/alspac/researchers/research-ethics/). Data were collected during clinic visits or with postal questionnaires. Please note that the study website contains details of all the data that is available through a fully searchable data dictionary and variable search tool at http://www.bristol.ac.uk/alspac/researchers/our-data/. For the purpose of our analysis, we included children of 8.5 years old (an age closer to the outcome measure in the MAVAN cohort), whose mothers had a pregnancy duration between 37 and 42 weeks, a maternal age at delivery greater than 18 years, a child birthweight greater than 2 kg, child alive at 1 year of age, and we only included singleton pregnancies in the analysis. Figure 1 describes the subset of the sample for the purpose of the analyses in the ALSPAC cohort. There were 467 subjects with complete data available for the analyses.

#### Maternal Adversity, Vulnerability and Neurodevelopment Project

**FIGURE 2:**
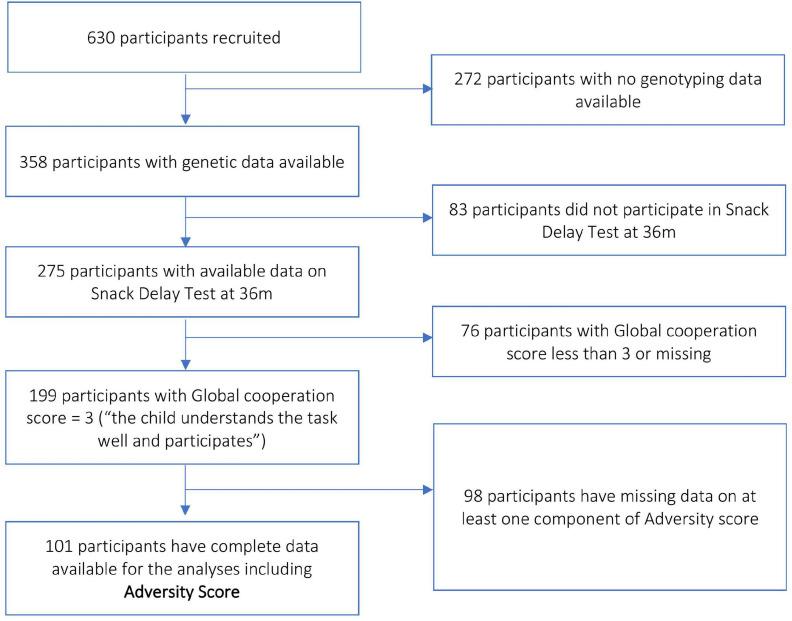
Block scheme describing sample selection in the MAVAN cohort.

The study MAVAN is a birth cohort that followed up children from birth up to 6 years of age in Montreal (Quebec) and Hamilton (Ontario), Canada, and has 630 recruited participants (O’Donnell et al., 2014). Mothers aged 18 years or above, with singleton pregnancies, and fluent in French or English were included in the study. Several maternal chronic illnesses, including placenta previa and history of incompetent cervix, impending delivery, a fetus/infant affected by a major anomaly, or gestational age < 37 weeks composed the exclusion criteria. Approval for the MAVAN project was obtained by the ethics committees and university affiliates (McGill University and Université de Montréal, the Royal Victoria Hospital, Jewish General Hospital, Centre hospitalier de l’Université de Montréal and Hôpital Maisonneuve-Rosemount) and St. Joseph’s Hospital and McMaster University, Hamilton, QC, Canada. Informed consent was obtained from all participants. Figure 2 describes the criteria and selection of MAVAN sample for the purpose of our research. There were 101 subjects with complete data available for the analyses.

### Genotyping

#### Avon Longitudinal Study of Parents and Children

Children in the ALSPAC cohort were genotyped using the Illumina HumanHap550 quad chip genotyping platform by the Wellcome Trust Sanger Institute, Cambridge, United Kingdom and the Laboratory Corporation of America, Burlington, NC, United States (Richmond et al., 2017). Standard quality control (QC) procedure was applied: participants with inconsistent self-reported and genotyped sex, minimal or excessive heterozygosity, high levels of individual missingness (>3%), and insufficient sample replication (IBD < 0.8) were excluded. Also, SNPs with call rate < 95%, MAF < 1%, or not in Hardy-Weinberg Equilibrium (HWE; *p* < 5 × 10^–7^) were removed. Following the QC, the genotyping data was imputed using Impute v3 and Haplotype Reference Consortium (HRC) imputation reference panel (release 1.1), which resulted in 38,898,739 SNPs available for analysis.

The population structure of ALSPAC cohort was described using principal component (PC) analysis (Patterson et al., 2006; Price et al., 2006), which was conducted on the genotyped SNPs with MAF > 5% with the following pruning parameters for linkage disequilibrium: 100-kilobase sliding window, an increment of 5 SNPs, and variance inflation factor (VIF) threshold of 1.01. To account for population stratification, the first ten PCs were included in the analysis.

#### Maternal Adversity, Vulnerability and Neurodevelopment

Genome-wide platforms (the Infinium PsychArray v1 or the PsychChip v1.1/v1.2, Illumina, Inc.) were used to genotype 229,456 autosomal SNPs of buccal epithelial cells of children in MAVAN, according to the manufacturer’s guidelines. SNPs with call rate < 95%, MAF < 5%, or not in HWE (*p* < 1 × 10^–30^) were removed. Afterward, imputation using the Sanger Imputation Service (McCarthy et al., 2016) and HRC as the reference panel (release 1.1) was performed and SNPs with an info score > 0.80 were retained for the analysis, resulting in 16,249,769 autosomal SNPs.

Similar to the ALSPAC cohort, the population structure of the MAVAN cohort was evaluated using PC analysis of all autosomal SNPs that passed the QC and not in high linkage disequilibrium (*r*^2^ > 0.2) across 50-kilobase region and an increment of 5 SNPs (Price et al., 2006). Based on the inspection of the scree plot, the first three PCs were the most informative of population structure and were included in all subsequent analyses.

### Polygenic Risk Scores

The rPRS procedure was administered in this study to inspect the interaction between genetic markers for fasting insulin and ELA to predict impulsivity in children using a GWAS constructed from adult data.

#### Avon Longitudinal Study of Parents and Children

**FIGURE 3:**
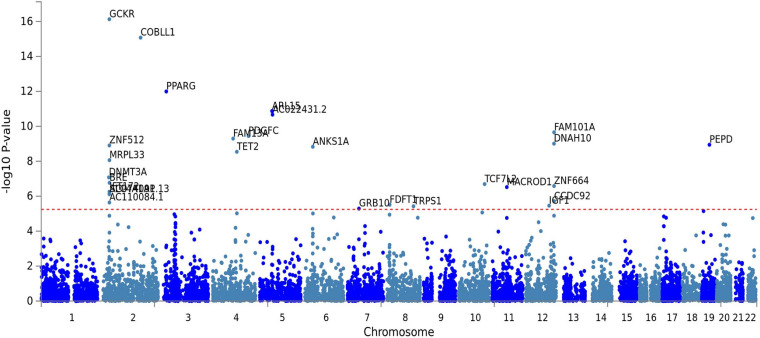
Genome-wide association study (GWAS) for fasting insulin from the Meta-Analyses of Glucose and Insulin-related traits Consortium (MAGIC). Each dot represents a single nucleotide polymorphism (SNP), with the *x*-axis showing genomic location and the *y*-axis showing the association level of each respective loci to fasting insulin. The gene names for all significant SNPs are displayed in the plot. This plot was obtained from FUMA.

**FIGURE 4:**
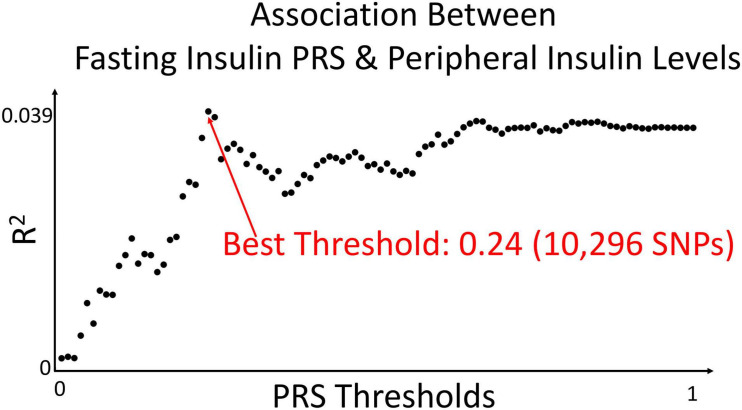
Association between fasting insulin PRS and peripheral insulin levels. We calculated polygenic risk scores from the fasting insulin GWAS at 100 different thresholds. Using ALSPAC as a discovery cohort, we identified the PRS threshold with the strongest correlation in predicting peripheral insulin levels in children at age 8.5 years. The strongest (R^2^ = 0.039) and most significant (*p* = 0.071) association was identified to be 0.24 in children at age 8.5 years in the ALSPAC cohort [*N* = 467; *p*_*t–initial*_ = 0.24 (10,296 SNPs)].

**FIGURE 5:**
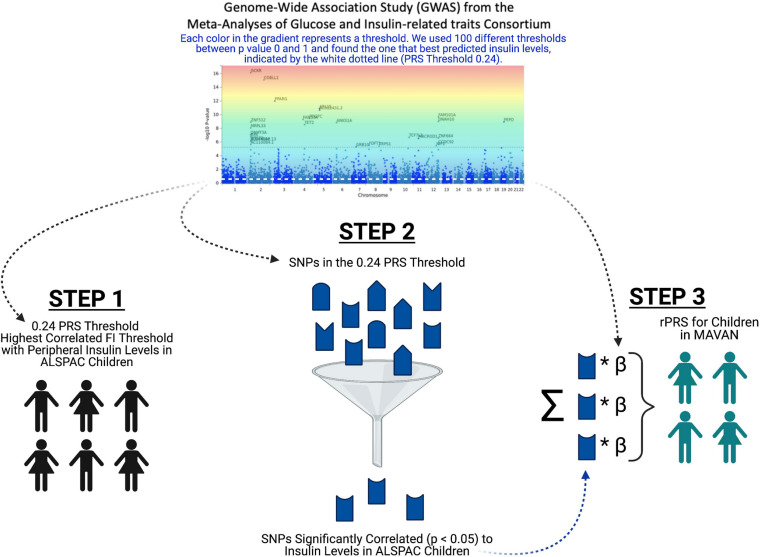
Refined PRS (rPRS) methodology. This flow chart depicts the rPRS process. Each color in the gradient on the fasting insulin GWAS represents a threshold. In step 1, we used 100 different thresholds between *p*-value 0 and 1 from the fasting insulin GWAS and found the threshold that best predicts insulin levels in ALSPAC Children (PRS threshold 0.24), indicated by the white dotted line on the GWAS. In step 2, we ran a correlation for each SNP within the 0.24 PRS threshold to find which SNPs significantly predicted (*p* < 0.05) the peripheral insulin levels. Lastly, in step 3, using the SNPs that significantly predicted peripheral insulin levels from step 2, we calculated the rPRS in the MAVAN cohort.

The fasting insulin PRS was calculated using the fasting insulin GWAS, shown in Figure 3 (*N* = 108,557) from the Meta-Analyses of Glucose and Insulin-related traits Consortium (MAGIC) (Scott et al., 2012). Prior to any PRS calculation, the GWAS was subjected to LD clumping with *r*^2^ of 0.2 and ALSPAC cohort as a reference dataset. PRS at 100 different GWAS *p*-value thresholds were calculated for each subject in the ALSPAC cohort as a sum of the risk alleles count weighted by the effect size described in the GWAS for each SNP (Dudbridge, 2013; Wray et al., 2014). Using ALSPAC as a discovery cohort, we identified the threshold at which the PRS had the best prediction of peripheral insulin levels in children at age 8.5 years. The strongest (R^2^ = 0.039) and most significant (*p* = 0.071) association in children at age 8.5 years was identified to be with a PRS at *p*_*t*–initial_ = 0.24 threshold (consisting of 10,296 SNPs) as shown in Figure 4. To further refine the PRS, a process explained through Figure 5, we ran a linear regression analysis for each SNP within the 0.24 threshold PRS to find which SNPs were significantly associated (*p*_*t*–refined_ < 0.05) with the peripheral insulin levels. There were 57 SNPs significantly associated with peripheral insulin levels within the SNPs included in the 0.24 threshold. The list of these SNPs can be found in Table 1 with their corresponding *p*-values from the fasting insulin GWAS (Scott et al., 2012). These 57 SNPs included in the rPRS were ranging in *p*-values from 0.000123 to 0.238 in the original GWAS (Scott et al., 2012), however, they were all significantly associated with the peripheral insulin levels in children (all *p*-values < 0.05). This finding confirms that SNPs associated with adult risk for high fasting insulin may not be the same as SNPs associated with children risk for high fasting insulin.

**TABLE 1:** Single nucleotide polymorphisms (SNPs) included in the refined PRS for MAVAN.

**SNP**	***P*-value (Fasting Insulin GWAS)**
rs7574670	0.000123
rs13225097	0.000846
rs196808	0.005017
rs6885750	0.010573
rs4841679	0.011172
rs870870	0.018874
rs2665316	0.019226
rs4405319	0.031478
rs11724118	0.045920
rs10804992	0.046243
rs6552502	0.058479
rs397234	0.058594
rs11898925	0.061942
rs1866816	0.061986
rs7807790	0.062096
rs2965106	0.062112
rs275146	0.062816
rs7155790	0.065848
rs9840453	0.067170
rs11693862	0.073821
rs1492377	0.074815
rs1377315	0.083513
rs4686837	0.088538
rs4803789	0.089959
rs10520768	0.090182
rs2295308	0.093524
rs7598551	0.094168
rs7332334	0.101271
rs728586	0.103540
rs4779876	0.103930
rs1935492	0.103989
rs7983099	0.112130
rs923554	0.118880
rs1885414	0.123295
rs9863801	0.126196
rs11891202	0.135653
rs4382157	0.136635
rs5753103	0.150747
rs17035960	0.151128
rs884972	0.151753
rs10002944	0.154237
rs10145606	0.156256
rs892114	0.159405
rs6543408	0.178625
rs4766912	0.184907
rs7138803	0.185122
rs2544164	0.197849
rs12219445	0.198058
rs1664256	0.199221
rs11603179	0.201429
rs11820303	0.211331
rs2341647	0.217823
rs6888754	0.219617
rs9808140	0.221567
rs1058065	0.223190
rs12731669	0.231006
rs7243066	0.238047

#### Maternal Adversity, Vulnerability and Neurodevelopment

The 57 SNPs within the PRS that we discovered to be associated with peripheral insulin levels in the ALSPAC cohort were used to construct a PRS in MAVAN. The PRS was standardized. Since the SNPs were selected through a refinement process of a PRS that was created through conventional means, we henceforth refer to this PRS in the MAVAN cohort as the rPRS. The rPRS was calculated similarly to the PRS scores in ALSPAC, as a weighted sum of 57 SNPs.

### Early Life Characterization

To investigate the interaction between the PRS and ELA, we estimated adversity exposure using a cumulative score involving different environmental variables (Silveira et al., 2017) for each individual in the MAVAN cohort as described by Silveira et al. (2017) and de Lima et al. (2020). The adversity score was created by combining several markers of adversity. The following instruments were included as markers in the score: (1) *The Health and Well-Being Questionnaire* (Kramer et al., 2001) to obtain information on how often and to what degree the woman lacked money for basic needs (Kanner et al., 1981) using the *Daily Hassles Scale*, on chronic stress with the romantic partner (Pearlin and Schooler, 1978), on conjugal violence (Newberger et al., 1992; Parker et al., 1993), on anxiety during pregnancy (Dunkel-Schetter, 1998), on birth size percentile, and on gestational age; (2) *Smoking during pregnancy*; (3) *Household gross income* (Daveluy et al., 1998); (4) *Child Health Questionnaire* (Plante et al., 2002) to assess acute, chronic conditions and hospitalizations; (5) *Maternal mental health* through the Beck Depression Inventory (BDI) (Beck and Ward, 1961), Edinburgh Postnatal Depression Scale (EPDS) to screen for postpartum depression (Cox et al., 1987), and State-Trait Anxiety Inventory (STAI) to measure psychological components of state and trait anxiety (Spielberger, 2010); (6) *Attachment* through the Preschool Separation-Reunion Procedure (PSRP) applied at 36 months (Cassidy et al., 1992) (Solomon and George, 2008); (7) *Family Assessment Device* to assess family functioning based on the McMaster Model of Family Functioning (Moss et al., 2004). For every item with a continuous score, we used either the 15th or the 85th percentile as the cut-off to add a point to the adversity score. Presence of each component yields one point, and the adversity score represents the summation of the points where the higher the score, the more adversity has been experienced by the individual.

### Behavioral Outcomes

In the Snack Delay Task at 36 months (Golden et al., 1977; Campbell et al., 1982; Vaughn et al., 1984; Kochanska et al., 1996), the children placed their hands flat on a table in front of them and restrained themselves from eating a single M&M candy from under a glass cup placed on the table. The children were told to delay eating until the research assistant rang a bell. The test was conducted over four distinct trials (using delays of 10, 20, 15, and 30 seconds). Halfway through each trial delay, the experimenter lifted the bell but did not ring it. The children received a behavioral score for each trial (“behavior code”) based on attempts to eat the candy before the bell rang. Coding ranged from 1 to 7, as displayed in Table 2. For each trial, the ability of the children to wait for the M&M was also recorded (snack delay latency to eat: 1 = child keeps hands on the table during the entire time either before OR after the bell is lifted and 2 = child keeps hands on the table during the entire time before AND after the bell is lifted). This latter score (1 or 2) and the behavioral code score (ranging from 1 to 7) culminated to provide a total performance score (ranging from 2 to 9) for each of the four trials. A “global cooperation score” rated the ability of the child to engage and complete the task (0 = the child is unwilling or unable to engage in the task; 1 = the child is unwilling or unable to complete the task because of feeling tired, angry, irritable, or sick, or does not have the capacity to understand the instructions; 2 = the child does all the trials but has comprehensive or motivational difficulties, or is passive or inhibited, and 3 = the child understands the task well and participates). Children with a global score of 3 were included in the analysis. The Snack Delay Task was applied to children in the MAVAN cohort at the age of 36 months.

**TABLE 2:** The Snack Delay Task was performed at 36 months in the children from the MAVAN cohort.

**Snack Delay – Test Scoring**	
1	Eats before bell is lifted
2	Eats after bell is lifted
3	Touches candy before bell is lifted
4	Touches candy after bell is lifted
5	Touches cup or candy before bell is lifted
6	Touches cup or candy after bell is lifted
7	Waits for bell to ring before touching cup or candy

*Each child was asked to place their hands flat on a table and to restrain themselves from eating a single M&M candy from under a glass cup placed on the table in front of them. The children were instructed to delay eating until the research assistant rang a bell. The test was conducted over four distinct trials (using delays of 10, 20, 15, and 30 seconds) and the scores of each trial were added together for the final cumulative score.*

### Statistical Analysis

Data analyses were carried out using R (R Core Team, 2019). Baseline comparisons between low and high PRS groups were done in the MAVAN cohort. Mean differences of the main confounding variables were assessed using the Student’s *t*-test for independent samples if they were continuous variables or the chi-square test if they were categorical variables. Significance levels for all measures were set at α < 0.05.

We used linear regression models to test the association between the outcome of impulsivity, evaluated using the Snack Delay Task in this case, and the predictors including adversity exposure, rPRS, and their interaction term with sex and the first three genomic PCs as covariates. In summary, we ran the following three linear regression models:

1. Outcome ∼ sex + PC1 + PC2 + PC3 + Adversity Score
2. Outcome ∼ sex + PC1 + PC2 + PC3 + rPRS
3. Outcome ∼ sex + PC1 + PC2 + PC3 + Adversity Score × rPRS

To identify the form of interaction between the rPRS and the adversity score, we used Roisman’s method (Roisman et al., 2012; Belsky et al., 2015) of simple slopes analysis and examined the regions of significance (RoS) to determine the range of values of the predictor for which regression of the outcomes on the moderator (rPRS) is statistically significant. To explore the form of interaction, Roisman also recommends the use of two metrics designed to help identify between diathesis-stress and differential susceptibility models: the proportion of interaction (PoI) index and the proportion affected (PA) or the percentage above index. Both metrics show a preponderance of differential susceptibility when greater than a certain threshold. As a sub-analysis to handle the missing cases for the adversity score, we imputed the data with hot-deck imputation (hot.deck package in R) (Cranmer et al., 2020), assuming missing at random mechanisms. We imputed all adversity score components, calculated the extended adversity score on an additional 98 subjects, and repeated the linear regression analysis on the imputed datasets of 199 subjects each, reporting the pooled estimates from 30 imputed sets.

### Enrichment Analysis

Enrichment analyses for gene ontologies were performed using MetaCore^TM^ (Clarivate Analytics) on the SNPs that compose the fasting insulin rPRS. Furthermore, gene-based enrichment analyses were performed in FUMA^1^ (MacArthur et al., 2017; Watanabe et al., 2017; Aguet et al., 2019) after mapping the SNPs composing the fasting insulin rPRS to genes with the biomaRt package in R (Durinck et al., 2005, Durinck et al., 2009). We also used GeneMANIA (Warde-Farley et al., 2010) to determine if the genes were part of a network. Specifically, the gene list derived from the fasting insulin rPRS is entered in GeneMANIA. GeneMANIA then extracts linked mRNA expression data from the Gene Expression Omnibus (GEO) and connects co-expressed data to form functional association networks. The node sizes represent gene scores indicating the number of paths that start at a given gene node and end up in one of the query genes.

## Results

### Baseline Characteristics

Baseline comparisons between low and high rPRS groups were performed in the MAVAN cohort. No differences were found for the main confounding variables in the MAVAN cohort, as shown in Table 3. Participants’ characteristics for ALSPAC cohort are reported in Table 4. Table 5 details the degree of missing data for each component of the adversity score.

**TABLE 3:** Participants’ characteristics in MAVAN.

**Sample descriptive**	**Total (*n* = 101)**	**Low PRS (*n* = 50)**	**High PRS (*n* = 51)**	***p***
Sex – male	44.6% (45)	42.0% (21)	47.1% (24)	0.756
Maternal age at birth (years)	30.51 (4.65)	30.17 (4.36)	30.84 (4.94)	0.470
Gestational age (weeks)	39.32 (1.17)	39.34 (1.27)	39.29 (1.08)	0.846
Birth weight (g)	3,326 (458)	3,371 (472)	3,281 (443)	0.325
Duration of breastfeeding (months)	7.23 (4.82)	6.91 (4.83)	7.54 (4.83)	0.511
Smoking during pregnancy	11.9% (12)	10.0% (5)	13.7% (7)	0.786
Maternal education – university degree or above	62.4% (63)	68.0% (34)	56.9% (29)	0.342
Low income at 36 m	11.2% (11)	12.2% (6)	10.2% (5)	1.000
Self-reported ethnicity (Caucasian)	78.1% (75)	80.9% (38)	77.1% (37)	0.2

*Numbers are presented as mean (SD) or percentage (number of participants). Comparison between low/high PRS groups were carried out using Student *t*-test for continuous variables and chi-square test for categorical variables.*

**TABLE 4:** Participants’ characteristics in ALSPAC.

**Sample descriptive**	**Total (*n* = 467)**
Sex – male	52.5% (245)
Maternal age at birth (years)	29.89 (4.44)
Gestational age (weeks)	39.76 (1.18)
Birth weight (g)	3,532 (482)
Breastfeeding at 3m (yes)	51.5% (240)
Smoking during pregnancy (yes)	19.1% (89)
Maternal education – university degree or above	18.7% (84)
Low Socioeconomic Status (SES) measured at 2 years, 9 months	37.3% (174)
Self-reported ethnicity (White)	99.8% (457)

*Numbers are presented as mean (SD) or percentage (number of participants).*

**TABLE 5:** Details of missing data for specific components of the adversity score in Maternal Adversity, Vulnerability and Neurodevelopment (MAVAN).

**Instruments included in the Adversity score**	**Number of items**	***N* available**	**Missing**
Birth information	2	199	0
Household gross income	1	199	0
Child hospitalization	1	199	0
Family Assessment Device	12	136	63
Attachment (scoring 28 min video of parent-child interaction)	1	165	34
Beck Depression Inventory	21	170	29
Edinburgh Postnatal Depression Scale	10	182	17
State-Trait Anxiety Inventory	40	195	4
Pregnancy anxiety	1	172	27
Smoking during pregnancy	1	172	27
Marital strain	9	193	6
Daily Hassles Scale	5	199	0
Physical/sexual abuse	2	199	0
Adversity score	Composite	101	98

*For the main analysis, only complete cases were considered. A sub-analysis after imputing the missing variables using hot deck imputation was also performed.*

### Interaction Between Fasting Insulin PRS and the Adversity Score Associates With Impulsivity in MAVAN

**FIGURE 6:**
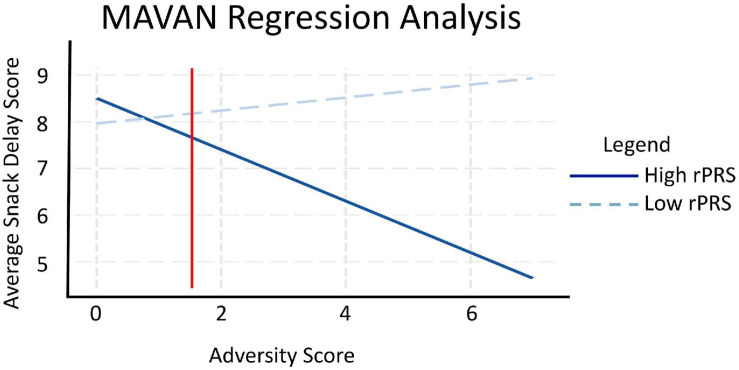
Maternal Adversity, Vulnerability and Neurodevelopment regression analysis. rPRS was calculated for children in MAVAN using the fasting insulin GWAS with significant SNPs identified in ALSPAC through the rPRS methodology. Linear regression analysis showed a significant interaction between fasting insulin rPRS and adversity exposure on impulsivity [*N* = 101, β = –0.329, *p* = 0.024], tested by the Snack Delay Task applied at 36 months: higher adversity is linked to higher impulsivity in children with higher rPRS [rPRS = mean ± 1 SD, β = –0.551, *p* = 0.009], represented by the solid blue line. The dashed light blue line represents the low rPRS group, where the effect of adversity on impulsivity using the Snack Delay Task was not significant [β = 0.139, *p* = 0.348]. We also analyzed the form of these interactions according to Roisman. The regions of significance, as well as the proportion of interaction (PoI = 0.984) and proportion affected (PA = 0.782) are consistent with the diathesis stress form of interaction.

We performed a linear regression analysis to investigate the interaction effect between the refined genetic score (rPRS) and adversity exposure on the Snack Delay Task in the MAVAN cohort applied at 36 months, adjusted by population stratification PCs and sex. A significant interaction effect was observed, as displayed in Figure 6, between fasting insulin rPRS and adversity exposure on impulsivity measured by the Snack Delay Task [β = −0.329, *p* = 0.024]. Simple slope analysis at ± 1 SD rPRS showed that higher ELA is linked to more impulsivity in children with higher rPRS [β = −0.551, *p* = 0.009]; there was no effect of adversity on impulsivity in the low rPRS group [β = 0.139, *p* = 0.348]. The region of significance is to the right side of the red line in Figure 6, which suggests that the association between impulsivity and the rPRS is significant in children highly exposed to adversity. We also analyzed the form of the interaction according to Roisman (Roisman et al., 2012). The RoS, as well as the PoI (0.984) and PA (0.782) are consistent with the diathesis-stress model.

To obtain a distribution of the statistics of interest (interaction coefficient) and its confidence interval, we applied a non-parametric bootstrap, which resulted in estimated beta = −0.329 (SE = 0.1764) and 95% confidence interval (−0.6729, −0.0202) for the effect of interaction between rPRS and adversity on Snack Delay Task in MAVAN.

The main effect of the refined genetic score (rPRS) on the Snack Delay Task in MAVAN [*N* = 101] applied at 36 months, adjusted by PCs and sex, was not significant [β = −0.190, *p* = 0.126]. The main effect of adversity on the Snack Delay Task in MAVAN [*N* = 101] applied at 36 months, adjusted by PCs and sex, was also not significant [β = −0.131, *p* = 0.171]. When performing imputations on the missing cases of the adversity score (final *N* = 199), the effect of the interaction between rPRS and adversity score on Snack Delay outcome was no longer statistically significant [β = 0.082, *p* = 0.168].

### Enrichment Analysis on Fasting Insulin PRS

**FIGURE 7:**
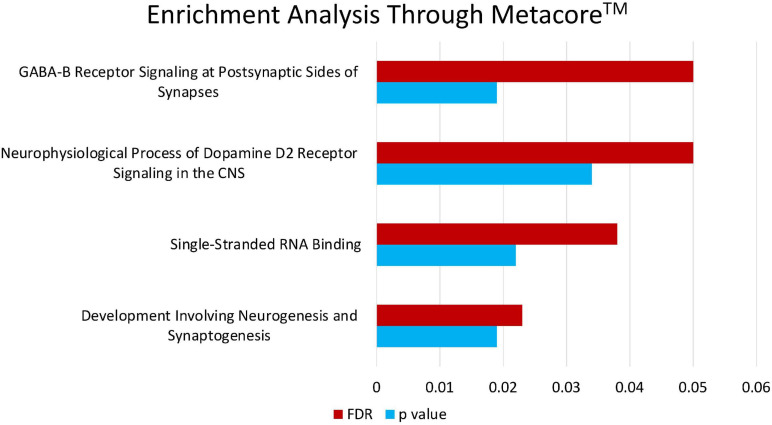
Enrichment analysis through Metacore^TM^. Enrichment analysis of the SNPs that compose the fasting insulin rPRS shows that this subset of SNPs is significant for certain nervous system development processes: neurophysiological process of GABA-B receptor signaling at postsynaptic sides of synapses (*p* = 0.019, FDR = 0.05), neurophysiological process of dopamine D2 receptor signaling in the CNS (*p* = 0.034, FDR = 0.05), single-stranded RNA binding (*p* = 0.022, FDR = 0.038), and development involving neurogenesis and synaptogenesis (*p* = 0.019, FDR = 0.023).

**FIGURE 8:**
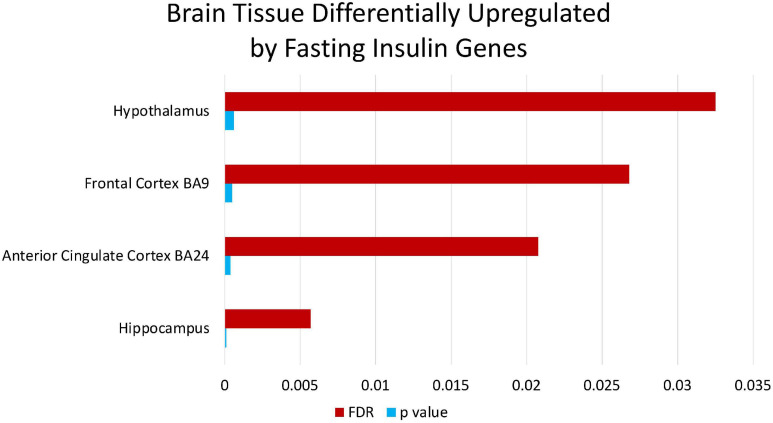
Brain tissue differentially upregulated by fasting insulin genes. Enrichment analysis through FUMA of the genes mapped by the SNPs that compose the fasting insulin rPRS show that these genes are significantly differentially upregulated in brain specific tissues identified in the figure.

**FIGURE 9:**
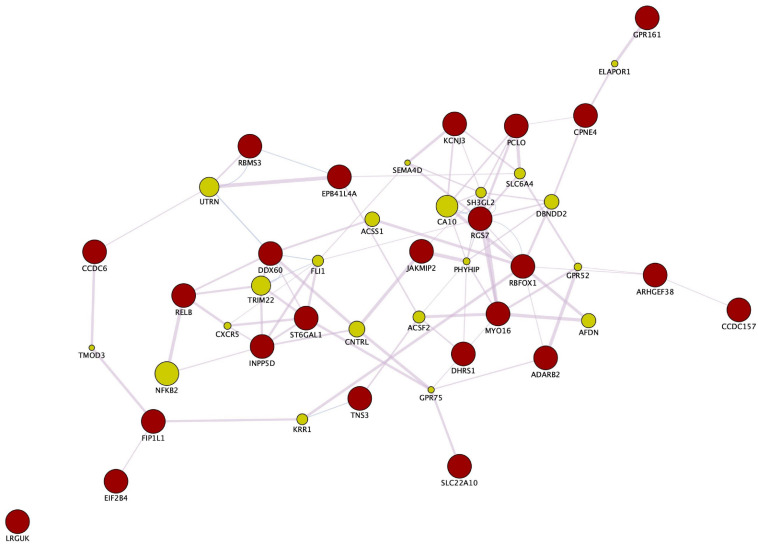
Gene co-expression network. Enrichment analysis through GeneMANIA and Cytoscape of the genes mapped by the SNPs that compose the fasting insulin rPRS show that these genes are part of a co-expression network in *Homo sapiens*. Red circles indicate query genes, whereas green circles indicate related genes added by GeneMANIA. GeneMANIA translates mRNA expression data from Gene Expression Omnibus (GEO) to functional association networks that connect co-expressed genes through the pink lines displayed. The node sizes represent gene scores, indicating the number of paths that start at a given gene node and end up in one of the query genes.

Enrichment analyses (MetaCore^TM^) of the SNPs that compose the fasting insulin refined genetic score (rPRS) show that this subset of SNPs was significant for several nervous system development processes, as shown in Figure 7. Enrichment analysis (FUMA, see text footnote 1) of the genes mapped by the SNPs that compose the fasting insulin rPRS showed that these genes were significantly differentially upregulated in the following brain specific tissues, as shown in Figure 8: hippocampus, frontal cortex Brodmann area 9 (BA9), anterior cingulate cortex Brodmann area 24 (BA24), and the hypothalamus. Furthermore, these genes also had a significant GWAS enrichment for accelerated cognitive decline after conversion of mild cognitive impairment to Alzheimer’s disease (*FDR adjusted p-value* = 0.013). Using GeneMANIA (Franz et al., 2018), we discovered that this set of genes was part of a single co-expression network in *Homo sapiens*, as shown in Figure 9, indicating their shared involvement in biological processes (Ma et al., 2018).

## Discussion

The purpose of this study was to explore whether the genetic background associated with higher fasting insulin interacts with ELA to predict impulsivity, tested using the Snack Delay Task, in children. We demonstrated that the calculation of a rPRS, consisting of SNPs most associated with peripheral insulin levels in children and representing the risk for high fasting insulin levels early in life, can be derived from the GWAS of fasting insulin in adults. This refined polygenic score interacted with ELA exposure to predict impulsivity in children in the MAVAN cohort. Additionally, we observed that the SNPs composing the fasting insulin rPRS and their mapped genes were significantly correlated with various nervous system development processes.

Instead of using an arbitrary *p*-value threshold to calculate the PRS, we calculated the PRS at one hundred different thresholds in the independent cohort ALSPAC as a training sample. To calculate the PRSs, we applied the PRSoS tool (Chen et al., 2018). For each threshold, we explored the association between PRS and peripheral insulin levels within the ALSPAC cohort. This technique allowed us to identify the threshold of 0.24 to best predict peripheral insulin levels in children (Chen et al., 2020). To further refine this PRS, we associated each SNP within the subset obtained from the 0.24 threshold PRS with peripheral insulin levels in the discovery cohort ALSPAC and selected only the SNPs that significantly predicted the peripheral insulin levels in children to calculate our final rPRS. This refinement was necessary as the genetic markers for fasting insulin levels in adults may not be comparable to the genetic markers for fasting insulin levels in children. Since there is no GWAS available to identify the SNPs most associated with risk for high fasting insulin levels in children, we took an alternative approach, the rPRS, that allowed us to identify a subset of SNPs associated with high fasting insulin levels in children. Usually, analyses identifying which PRS threshold should be used are based on the greatest proportion of variance explained in the outcome, which would be the Snack Delay Task in this study. That does not take into account which PRS threshold is best predicting the phenotype composing the PRS itself, making our approach distinctive. Another strength in our approach is that we used a training sample to identify the best PRS threshold to use in our test sample. Subsetting the list of SNPs further adds to our distinctive methodology because we can be confident that the genetic background of fasting insulin used within the analysis is in fact correlated with actual peripheral insulin levels in children. The rPRS is a better predictor than peripheral insulin levels because the genetic background represents a more stable characteristic than the fluctuant insulin levels, which oscillate diurnally and may not be an accurate representation of a child’s fasting insulin levels later in adulthood. By using the rPRS, which was calculated using the GWAS related to adult fasting insulin levels, we obtained a more accurate representation of the risk of a child to develop high fasting insulin. There is no overall effect of either adversity score or rPRS, but there is an interaction effect of adversity and rPRS on impulsivity at 36 months. Specifically, the effect of adversity on impulsivity was seen for individuals with a higher rPRS for fasting insulin.

Although the results of the interaction between rPRS and adversity were no longer significant after imputing the missing cases for the adversity score, we want to emphasize that the adversity score is a composite measure computed based on several different tools and assessments, including total scores of instruments (e.g., BDI) or complex behavioral tasks such as the Attachment profile assessed through detailed coding of filmed interactions (Strange Situation Task) (Table 5). Therefore, as much as imputations can be technically performed, we are not convinced that the imputed data can capture the multifaceted feature of our unique composite score, so our main analysis is focused on complete cases. Our missing data was mostly related to unit-level non-response (no information was collected for the respondent on a specific survey/instrument/questionnaire) rather than item non-response (the respondent was missing one or two questions of the survey/instrument/questionnaire) (Dong and Peng, 2013). Unit-level non-response can be more challenging to impute with confidence (Yan and Curtin, 2010). Relaxing the complexity of the adversity score mentioned above and considering the missing components of the score as missing items in the dataset, the hot-deck imputation was applied. While imputation is useful and necessary to support analysis and summarization, the imputation model should be properly specified, which we believe is difficult to achieve in this particular case. Some of the variables that compose the adversity score, for example the Attachment security information, are derived from a laboratory procedure designed to capture the balance of attachment and exploratory behavior under conditions of increasing moderate stress (Solomon and George, 1999), a unique measure that is hardly comparable to any other measure available in the dataset. Finally, we assumed a missing at random mechanism for the missing data, although adversity itself could be associated with the missingness pattern (Howe et al., 2013; Houtepen et al., 2018). Therefore, the results of this sub-analysis should be considered with caution.

The gene ontology enrichment analyses, done through MetaCore^TM^, showed that the SNPs composing the fasting insulin rPRS are associated with nervous system development. Some of the enriched processes should be highlighted, such as the neurophysiological process of dopamine D2 receptor signaling in the central nervous system. The dopamine system has been linked to impulsive behavior in animal models and human studies (van Gaalen et al., 2006; Dalley and Roiser, 2012). This finding is interesting because it suggests a potential neurodevelopment pathway to support the relationship between the genetic background linked to insulin, ELA, and dopamine. Previous animal models from our laboratory have demonstrated that animals exposed to ELA showed a pronounced aversion to delayed rewards in addition to an increase in the medial prefrontal cortex D2 levels (Alves et al., 2019). Additionally, our laboratory has shown that animals that have experienced ELA have a delay in dopamine release in the nucleus accumbens in response to palatable food, but insulin administration reverts this delayed effect (Laureano et al., 2019). These studies show that the relationship between ELA and dopamine is moderated by insulin. The set of fasting insulin SNPs identified in this present study could lead to further insight on the genetic background linking dopamine to impulsivity. Since the mechanism involving this association is still unknown, our findings bring us a step closer to this understanding.

Enrichment analyses in MetaCore^TM^ revealed that the rPRS is enriched for single-stranded RNA binding. This suggests that the SNPs composing the rPRS play a crucial role in post-transcriptional regulation of gene expression (Guo et al., 2014) and hence are key in gene by environment interaction effects. The genes mapped to the SNPs in the rPRS are also differentially upregulated in the frontal cortex BA9, which is known to be involved in several executive functions such as short-term memory, inductive reasoning, working memory, and planning (Yogev-Seligmann et al., 2008). These findings, in addition to the genes being enriched for the accelerated cognitive decline GWAS, suggest that the genetic background associated with fasting insulin can impact several neurodevelopment executive functions, impulsivity being one of them, as well as risk for cognitive decline later in life. This aligns with studies that identified insulin receptors at hippocampal glutamatergic synapses, suggesting a role of insulin in neurotransmission, synaptic plasticity, and modulation of learning and memory, while its inhibition is described in Alzheimer’s disease and related animal models (Bomfim et al., 2012).

There are limitations within our study. Our discovery cohort and our testing cohort both largely consist of White/European ancestry, allowing us to identify the SNPs required within one cohort and testing the hypothesis within another cohort that has a similar population structure. Unfortunately, we cannot be certain that this subset of SNPs will be relevant for a different ancestry. Different ancestries have distinct allele frequencies (Frudakis et al., 2003) and this could result in peculiarities in the interaction between the genetic background and the environment. In fact, differential linkage disequilibrium between ancestral populations can produce false-positive SNPs when local ancestry is ignored, meaning that gene expression traits have differences as a function of genetic ancestry (Park et al., 2018). Furthermore, several studies showed genomic differences when investigating multi-ancestry genomic analysis (Bryc et al., 2015; Sung et al., 2019). In addition to ancestry, culture can impact one’s behaviors, especially those related to executive functions like impulse control. There have been several examples of gene-culture interactions such as the cultivators in West Africa whose agriculture, which consisted of malaria-carrying mosquitos, showed preference for the hemoglobin S (*HbS*) “sickle-cell” allele to provide protection from malaria (Livingstone, 1958). Similarly, Polynesians being exposed to cold stress and starvation during their long open-ocean voyages may have resulted in positive selection for thrifty metabolism leading to type 2 diabetes susceptibility in present day Polynesians (Houghton, 1990). This gene-culture evolution emphasizes that one’s lifestyle and environment have lasting impact and could be responsible for the differences seen in gene-environment interactions. Unfortunately, to the best of our knowledge, there is currently no fasting insulin GWAS available in a different ancestry for us to address this limitation within our work. Future studies including a discovery cohort with peripheral insulin information in children and testing cohort of similar population structure in children are warranted.

These results together confirm that both ELA and the biological machinery associated with higher insulin levels are important factors influencing impulsivity in children. Our analyses showed that the genetic background associated with high fasting insulin levels moderates the effects of adversity on childhood impulsivity. This reinforces the idea that insulin signaling, which is implicated in metabolism and child growth, also plays a role in neurodevelopment. Previous studies have shown that impulsivity is a core feature of both psychopathology and metabolic diseases (Schachar and Logan, 1990; Silveira et al., 2012; Testa et al., 2019). Therefore, the interaction described here could be the basis to explain the co-morbidity associated with ELA exposure. Our results align with Hari Dass et al. (2019), which used a biologically-informed polygenic score based on insulin-related gene networks to predict both childhood impulsivity and risk for dementia later in life.

In conclusion, our present findings provide support for the impact of exposure to ELA in interaction with the genetic profile associated with high fasting insulin in predicting executive functions such as impulsivity in children. This research can be highly impactful as it provides insights into the vulnerability of executive function disorders early on in an individual’s life. The biological mechanisms that we discovered to be involved in these processes can inform the development of early interventions and more efficient management of such health outcomes.

## Data Availability Statement

The original contributions presented in the study are included in the article/Supplementary Material, further inquiries can be directed to the corresponding author.

## Ethics Statement

Ethics approval for the ALSPAC study was obtained from the ALSPAC Ethics and Law Committee and the local research ethics committees (a full list of the ethics committees that approved different aspects of the ALSPAC studies is available at http://www.bristol.ac.uk/alspac/researchers/research-ethics/). Ethics approval for the MAVAN project was obtained from obstetricians performing deliveries at the study hospitals and by the institutional review boards at hospitals and university affiliates: McGill University, l’Université de Montréal, the Royal Victoria Hospital, Jewish General Hospital, Centre Hospitalier de l’Université de Montréal, Hôpital Maisonneuve-Rosemont, St Joseph’s Hospital, and McMaster University, Hamilton, ON, Canada. Informed consent was obtained from the parents/guardians of the participants. Written informed consent to participate in this study was provided by the participants’ legal guardian/next of kin.

## Author Contributions

AB and PPS designed the experiments. AB, LMC, ZW, IP, and SP performed the analysis. LMC, CP, IP, RDL, MJM, and PPS provided important feedback and support for the data analysis and manuscript writing. AB wrote the first draft of the manuscript. All authors contributed to the article and approved the submitted version.

## Conflict of Interest

The authors declare that the research was conducted in the absence of any commercial or financial relationships that could be construed as a potential conflict of interest.

## Publisher’s Note

All claims expressed in this article are solely those of the authors and do not necessarily represent those of their affiliated organizations, or those of the publisher, the editors and the reviewers. Any product that may be evaluated in this article, or claim that may be made by its manufacturer, is not guaranteed or endorsed by the publisher.

## Footnotes

1
https://fuma.ctglab.nl/
