# Supplementary material for: Early Life Adversity and Polygenic Risk for High Fasting Insulin Are Associated With Childhood Impulsivity
Source: Front Neurosci. 2021 Sep 1;15:704785. doi: 10.3389/fnins.2021.704785 (PMC8441000; doi:10.3389/fnins.2021.704785)
Supplement: Supplementary file 1 [file Table_1.docx]

­Supplementary Material

Supplementary Table 1. Adversity Score: For each individual in the MAVAN cohort, an adversity score is calculated. Each item, except birth size, was binarized using either 15^th^ or 85^th^ percentile as the cut-off. Depending on the scoring system of the item, values below 15^th^ or values higher than 85^th^ percentile were considered as adversity, other values were considered as no adversity. The only item that includes both below 10^th^ as well as above 90^th^ percentiles as adversity is birth size (in other words, both low and high birth size were considered adversity). Presence of each component yields one point and the final postnatal score represents the summation of the points where the higher the score, the more adversity has been experienced by the individual.

| **Adversity Cumulative Score** |
| --- |
| Hospitalizations in the first 6 months of life |
| Birth Size |
| Gestational age below or equal to 37 weeks |
| Maternal mental health (BDI, EPDS, STAI) |
| Household total gross income |
| Lack of money score |
| Disorganized attachment |
| Poor family function (FAD) |
| Presence of domestic violence or sexual abuse |
| Presence of marital strain |
| Smoking during pregnancy |
| Pregnancy anxiety |
